# Knowledge Improvement After a Neonatal Resuscitation Course in a Low-Resource Setting

**DOI:** 10.3390/children13101286

**Published:** 2026-09-22

**Authors:** Giulia Merendi, Paolo Ernesto Villani, Amani Pokou Rebecca N’Guessan Kouame, Aya Georgette N’Goran Koffi, Anna Margherita Buriola, Francesco Cavallin, Laura Canonico, Giovanni Putoto, Daniele Trevisanuto

**Affiliations:** 1 Doctors with Africa CUAMM, Abidjan 002-14, Côte d’Ivoire; g.merendi@cuamm.org (G.M.); l.canonico@cuamm.org (L.C.); 2 Department of Women’s and Child Health, Fondazione Poliambulanza Hospital, 25124 Brescia, Italy; paolo.villani@poliambulanza.it; 3 Programme National de Santé Mère-Enfant, Ministère de la Santé, de l’Hygiène Publique et de la Couverture Maladie Universelle (MSHPCMU), Abidjan 002-03, Côte d’Ivoire; rebecca_kouame@yahoo.fr (A.P.R.N.K.); jorfiaya@gmail.com (A.G.N.K.); 4 Department of Woman’s and Child’s Health, University of Padua, 35128 Padua, Italy; annamargherita.buriola@studenti.unipd.it; 5 Independent Statistician, 36020 Solagna, Italy; 6 Doctors with Africa CUAMM, 35121 Padua, Italy; g.putoto@cuamm.org

**Keywords:** education, low-resource setting, newborn, resuscitation

## Abstract

**Highlights:**

**What are the main findings?**

• Overall, the Helping Babies Breathe (HBB) course increased neonatal resuscitation knowledge after the course.
• Performance was lower at the three-month assessment than immediately after the course.

**What are the implications of the main finding?**

• A low-dose high-frequency refreshing course may be offered to help participants maintaining the acquired knowledge.
• Further research including longer follow-up periods may assess the durability of training effects, identify the optimal timing for refresher training, and evaluate whether improved knowledge translates into better clinical practice and neonatal outcomes.

**Abstract:**

Background: The application of neonatal guidelines developed for high-resource settings is often difficult in low-resource settings due to structural differences and lack of equipment and personnel. The Helping Babies Breathe (HBB) program has been developed to overcome this limitation by adapting the protocols to local needs and resources. Objective: To evaluate knowledge after the HBB course among healthcare providers in a low-resource setting. Methods: A prospective, observational assessment of knowledge after a HBB course and knowledge retainment three months later. Participants were 38 healthcare providers in a referral hospital of Abidjan in Côte d’Ivoire. A multiple-choice written test was administered before the course, after the course and three months later to assess knowledge changes. Results: Over maximum 18 points, median total score was 14 points (IQR 12–15) before the course, 15 points (IQR 13–16) after the course, and 14 points (IQR 13–15) three months later. A passing criterion of ≥80% was achieved by 26% participants before the course, 58% after the course (*p* = 0.006) and 39% three months later (*p* = 0.07). Conclusions: In a referral hospital in Côte d’Ivoire, neonatal resuscitation knowledge of healthcare staff increased after a HBB course but the performance was lower at the three-month assessment than immediately after the course. Further research may assess long-term knowledge retainment and future educational interventions may target other areas of improvement.

## 1. Introduction

The 2025 report of the United Nations Inter-Agency Group for Child Mortality Estimation indicated approximately 4.9 million deaths in children under 5 years, with half of them occurring within the first 28 days of life [1]. Intrapartum-related complications are the second cause of neonatal mortality and most of them can be prevented or managed with quality care from antenatal to postnatal periods [1,2].

Sub-Saharan Africa accounts for the largest share of neonatal mortality, with 27 deaths per 1000 live births [3]. The 2015 Sustainable Development Goals of the United Nations set the target of lowering child mortality to under 12 per 1000 live births by 2030 [4]. Every year, approximately ten million newborns do not start breathing immediately after birth and require prompt assistance (“the Golden Minute”) to avoid neonatal mortality [4]. However, shortage of professionals and lack of formal education in neonatal resuscitation among birth attendants have a negative impact on neonatal outcomes in low-resource settings [4]. The World Health Organization estimated that countries would require approximately 44.5 health providers for every 10,000 people to meet the Sustainable Development Goal for health and well-being, but the 10 countries with the highest newborn mortality have around 11 health workers for every 10,000 people [5]. In additions, birth attendants are frequently not trained to provide fetal monitoring or newborn resuscitation, leading to delayed ventilation [6].

The application of neonatal guidelines that were developed for high-resource settings is often difficult in low-resource settings due to structural differences and lack of equipment and personnel. The Helping Babies Breathe (HBB) program has been developed to overcome this limitation by adapting the protocols to local needs and resources [7]. The strengths of the program include low cost, feasibility and flexibility that allows country-specific adaptations to meet local needs [8,9]. The program includes a simulation-based approach focusing on practical neonatal resuscitation skills, with additional educational materials such as checklists and questionnaires [7]. The literature showed that HBB contributed to improving knowledge and skills of health care providers and reducing neonatal mortality in low-resource settings [8]. Nonetheless, the retention of learned skills is acknowledged as a major challenge, suggesting the importance of recurrent training [10].

Côte d’Ivoire is one of the countries with the highest neonatal mortality, accounting for 27 deaths per 1000 live births in 2024 [11]. The World Health Organization found persistent inequalities in the coverage of healthcare services, as well as challenges in resource management and quality of care [12]. In 2025, an intervention was implemented in Abidjan (Côte d’Ivoire) to strengthen the quality of maternal and neonatal health. Among the dimensions of the intervention, healthcare providers were trained in neonatal care immediately after birth according to the HBB program.

## 2. Aim

We aimed to evaluate the knowledge after the HBB course and three months later among healthcare providers in a low-resource setting.

## 3. Materials and Methods

### 3.1. Study Design

This prospective assessment evaluated the knowledge after a HBB course and three months later among healthcare providers in a referral hospital of Abidjan in Côte d’Ivoire. The course was carried out within the framework of a broader maternal and neonatal health project.

### 3.2. Setting

The assessment was carried out at the maternity and neonatology departments of the Centre Hospitalier Régional Félix Houphouët-Boigny (CHR), located in the Abobo East health district of Abidjan (Côte d’Ivoire). The CHR is a secondary-level referral hospital covering more than 1.5 million people and accounting for approximately 7000 births annually. The CHR acts as a key facility for the management of neonatal and obstetric emergencies that cannot be addressed in primary healthcare facilities [13].

### 3.3. Participants

The participants were the 38 healthcare professionals who were involved in maternal and neonatal care at CHR, including 32 midwives and six clinicians working in the maternity and neonatology services.

### 3.4. Intervention

The course was carried out within the framework of a broader maternal and neonatal health project (protocol number 06346/MSHPCMU/CAB) that was implemented in the Abobo East health district of Abidjan (Côte d’Ivoire) by the University of Padova (Italy) and the Non-Governmental Organization Doctors with Africa CUAMM (Italy), in partnership with the Ministry of Health (Côte d’Ivoire), and was financed by the Italian Agency for Development Cooperation (AICS) with the support of the Italian Government [13].

The project entitled “Towards Universal Health Coverage: Strengthening the Quality of Maternal and Neonatal Health in Côte d’Ivoire” was launched in March 2025 with a total duration of 36 months. The interventions include infrastructural improvements, provision of modern equipment, development of standardized protocols for emergency identification and management, and training of healthcare providers [14]. In addition, acceptability of care and community engagement are implemented with awareness campaigns, education on maternal and neonatal health and involvement of the local community [14].

In September 2025, an educational course based on the Helping Babies Breathe (HBB) program was conducted in French by a multidisciplinary team comprising two Ministry of Health-certified neonatologists and two pediatricians, supported by three trained facilitators. The course followed the standard HBB program and aimed to provide participants with theoretical knowledge and practical skills for the recognition and management of newborns requiring resuscitation at birth. Emphasis was placed on timely assessment, initial resuscitation steps, effective positive-pressure ventilation, stabilization, and essential newborn care following resuscitation. The training consisted of three 4 h, face-to-face sessions delivered over two consecutive days and included three components: theoretical lessons, practical skill stations, and simulation exercises. Approximately 1.5 h were dedicated to interactive theoretical teaching covering the physiology and pathophysiology of birth, initial newborn assessment and management, resuscitation steps, and positive-pressure ventilation. Sessions also included manikin demonstrations, essential newborn care, and group discussion to facilitate the transition from theoretical knowledge to practical application. A further 1.5 h were devoted to supervised hands-on practice using neonatal manikins. Participants repeatedly practiced the initial resuscitation sequence and ventilation techniques, while facilitators provided immediate feedback and addressed technical difficulties. This approach was intended to promote familiarity with the equipment and procedural competence before simulation-based training. The remaining hour consisted of simulation exercises reproducing clinical situations requiring neonatal resuscitation and stabilization. Three scenarios were performed: a mildly depressed newborn, a severely asphyxiated newborn, and a preterm infant. Participants were required to promptly recognize the clinical condition, initiate appropriate resuscitation, and provide subsequent stabilization according to the HBB approach. Practical and simulation activities used four full-term neonatal manikins, including two NewBorn Anne and two NeoNatalie models (Laerdal, Stavanger, Norway), together with blankets, caps, suction pumps, and self-inflating bags. Standardized manikins allowed resuscitation procedures to be practiced in a controlled and consistent environment. The training was completed according to the predefined schedule, with all three educational components delivered as planned. Following the course, participants received no additional formal training, supervised practice, or refresher sessions before the three-month assessment. Thus, no structured HBB or neonatal resuscitation activities were undertaken during the interval between training and assessment.

### 3.5. Instruments

A multiple-choice written test was administered before and after the course to assess knowledge acquisition (September 2025), and three months later to assess knowledge retainment (December 2025). The test included 18 items (Supplementary Table S1) and was derived from the Knowledge Check (HBB 2nd Edition), which is a validated standardized questionnaire of the HBB program [15]. The questionnaire evaluates theoretical knowledge of neonatal resuscitation in low-resource settings, including preparation for birth, newborn evaluation, initial steps of birth asphyxia management, cord clamping, bag-mask ventilation, the importance of the “golden minute”, and step-by-step decision-making in neonatal resuscitation [15].

### 3.6. Statistical Analysis

Numerical data were summarized as median and interquartile range (IQR), while categorical data as absolute and relative frequency (percentage). At each timepoint (before the course, after the course and three months later), participant’s knowledge was scored by summing the number of correct answers in the 18 items, and the total score could range from 0 to 18 points. Effect sizes for score changes between consecutive timepoints were reported as median difference with 99% bootstrap confidence intervals. Due to coverage error of bootstrap confidence intervals for percentiles in small-sized groups, empirical bootstrap 99% confidence intervals were calculated using re-sampling with replacement (1000 samples of the same size as the original). A passing criterion of ≥80% (15 correct answers out of 18) was considered according to a previous study in Ethiopia [16], and pairwise comparisons between consecutive timepoints were performed using the McNemar exact test (function *mcnemar.exact* in package *exact2x2*). In addition, the number of correct answers for each item was reported at the three timepoints and compared between consecutive timepoints using the McNemar exact test (function *mcnemar.exact* in package *exact2x2*). All tests were two-sided and a *p*-value less than 0.05 was considered statistically significant. Adjustment for multiple testing was not applied given the exploratory (rather than confirmatory) purpose, and item-level comparisons were presented only as descriptive and hypothesis-generating. As subgroup analysis, the total score was stratified in clinicians and midwives with descriptive purpose and statistical testing was not performed due to the small subgroup size. The statistical analysis was carried out with R 4.6 (R Foundation for Statistical Computing, Vienna, Austria) [17].

## 4. Results

Thirty-eight health care providers (36 women and two men) attended the course and were assessed before the course, after the course and three months later. The sample included 32 midwives and six clinicians with a median 12 months (IQR 6–42) of experience in neonatal care.

Overall, the median total score was 14 points (IQR 12–15) before the course, 15 points (IQR 13–16) after the course, and 14 points (IQR 13–15) three months later (Figure 1). Median difference in score change from before to after the course was 1.0 points (99% bootstrap confidence interval −0.4 to 1.9 points). Median difference in score change from after the course to three months later was 0.0 points (99% bootstrap confidence interval −0.8 to 1.1 points).

The scatterplots in Figure 2 show how the total score of each participant changed from before to after the course, and from after the course to three months later. The individual waterfall plots in Figure 3 display the individual percent change in the total score over time.

Among the six clinicians, the median total score was 14 points (IQR 12–14) before the course, 16 points (IQR 16–17) after the course and 15 points (IQR 14–17) three months later. Among the 32 midwives, the median total score was 14 points (IQR 11–15) before the course, 14 points (IQR 13–15) after the course and 14 points (IQR 13–15) three months later.

Overall, a passing criterion of ≥80% was achieved by 10/38 participants (26%) before the course, 22/38 (58%) after the course (*p* = 0.004 compared to the previous assessment) and 15/38 (39%) three months later (*p* = 0.07 compared to the previous assessment). Figure 4 shows participants’ performance in the consecutive timepoints.

Figure 5 displays the number of correct answers for each item at the three timepoints (before the course, after the course and three months later). Statistically significant changes were observed only in routine care (from 63% before to 89% after the course, *p* = 0.01) and knowledge on newborn’s heart rate (from 37% after the course to 63% three months later, *p* = 0.01), while there were no statistically significant changes in the other items across the three timepoints. Item-level comparisons were presented only as descriptive and hypothesis-generating. There were no missing paired observations in the item-level analyses. Full numerical results are reported in Supplementary Table S2.

## 5. Discussion

Our report showed an overall knowledge improvement on neonatal resuscitation after a HBB course among healthcare providers in a referral hospital in Côte d’Ivoire, although the performance was lower at the three-month assessment than immediately after the course.

The course incorporated several components consistent with the Helping Babies Breathe and Helping Babies Survive educational frameworks [7]. Previous studies have established the efficacy of simulation-based training in acquiring neonatal resuscitation skills, where the trainees can apply theoretical knowledge in a controlled and safe environment [18,19]. Moreover, a multi-country study showed that knowledge improvement can be achieved by educational training activities irrespective of different healthcare systems, available resources and professional background [18].

The sequential goals of a neonatal resuscitation course include improvements of knowledge, manual skills and management of the scenario, followed by transfer on clinical practice. While the literature shows the clinical effectiveness of HBB course [8,9,20], we focused on knowledge improvement and decline that are the basis for building the clinical success of the program. Our findings contributed to the evidence about knowledge benefits after a HBB course, and confirmed that knowledge decline might occur even at short-term [8,21,22,23,24,25]. Moreover, they suggested that the minimal duration of the course (4 h) might provide limited benefits when baseline knowledge is low [7,16]. In low-resource settings, training programs on newborn resuscitation may result in common decline in performance over time, and skills deterioration may occur as early as 12 weeks after the initial training [24,25]. Previous studies recognized knowledge deterioration as one of the bottlenecks for long-term educational efficacy, and suggested low-dose high-frequency training as a potential approach for knowledge retention over time [23,25,26,27,28,29]. In these settings, the number of healthcare providers is often limited and resuscitation opportunities may be infrequent, hence routine practice of these skills is not always feasible. In our context, we believe that the involvement of local trainers and facilitators may have contributed to participant engagement and course acceptability [19,30]. Nonetheless, low-dose high-frequency training should be implemented to improve the long-term educational efficacy.

Beyond the overall knowledge improvement, the data might suggest some speculations about specific topics that could be targeted in future educational interventions. The number of correct answers regarding some topics (such as first minute priority, recognition of healthy newborn, consequences of unnecessary suctioning, bag-mask ventilation, newborn’s heart rate and post-resuscitation care) suggested that birth pathophysiology and bag-mask ventilation should require more attention during future training activities. These weak points may be mirrored by suboptimal practice in clinical setting [31]. On the other hand, topics such as thermal management, hygiene, routine care, cord management, care of depressed newborns, appropriate suctioning, stopping ventilation and device disinfection seemed to be well acquired during the course or consolidated in routine clinical practice.

Nonetheless, the impact of the HBB program on neonatal mortality may be limited by several factors that extend beyond the training of healthcare staff alone [8]. Although the acquisition of knowledge and practical skills in neonatal resuscitation represents an essential component of improving the management of birth asphyxia, training cannot fully address the structural and organizational barriers that may affect the timely provision of appropriate care [6,8]. Additional strategies are therefore needed to overcome persistent health system constraints, including staff shortages, which may limit both the participation of healthcare workers in training activities and their ability to provide adequate care during periods of high workload. Staff turnover represents another important challenge, as trained healthcare providers may subsequently leave their facilities or be transferred to other settings, resulting in a progressive loss of trained personnel and requiring repeated investments in training [6,8]. Furthermore, the effectiveness of neonatal resuscitation training may be compromised by the unavailability or inadequate maintenance of essential resuscitation equipment, as well as by poor infrastructure and limited access to basic resources. In such circumstances, even healthcare workers with adequate knowledge and skills may face difficulties in translating their training into effective clinical practice [6,8]. Another relevant issue is the high frequency of non-institutional deliveries in many low-resource settings. In these contexts, a substantial proportion of women may give birth outside healthcare facilities and therefore without the immediate support of trained healthcare providers or access to essential neonatal resuscitation equipment [5,6]. This situation is particularly concerning in cases of unexpected birth complications, when the rapid recognition of neonatal distress and the initiation of appropriate resuscitation measures are critical. In addition, when complications arise during or shortly after delivery, delays in reaching an adequately equipped health facility may further reduce the opportunity for timely intervention. Such delays can be related to inadequate referral systems, limited availability of transportation, long distances between communities and healthcare facilities, and poor road or communication infrastructure [5,6]. Consequently, improving neonatal outcomes is likely to require a comprehensive approach that combines healthcare worker training with broader health-system interventions, including the availability of adequately trained staff, essential equipment, functional referral pathways, and timely access to appropriate care. These complementary strategies may be particularly important for ensuring that the knowledge and skills acquired through programs such as HBB can be effectively translated into improved neonatal survival.

The present report has some limitations that should be acknowledged. First, the limited sample size reduced the capability of detecting some differences over time, and suggests caution in the interpretation of the findings. Moreover, it precluded any reasonable comparisons between subgroups of participants. Second, the single-center design limited the generalizability of the findings to similar settings, hence different knowledge improvement and retention may be found in other healthcare systems with different professional background and resource availability. For example, participants with low baseline knowledge may show the largest magnitude of improvement due to the ceiling imposed by the maximum score of 18, whereas participants with high baseline knowledge may improve the overall achievement of the passing criterion of ≥80%. Third, the follow-up assessment was restricted to three months, hence the data provide only limited information about the onset of knowledge decline. Long-term assessments are required to fully understand the durability of training effects and to identify the optimal timing for refresher training. Finally, only theoretical knowledge was assessed due to time constraints, whereas changes in practical skills or quality of care at birth in clinical practice could not be evaluated. The HBB program includes both theoretical and practical education, which should be assessed after the training [7]. Of note, theoretical knowledge and practical skills may follow different temporal trajectories, with practical skills declining earlier than theoretical knowledge [18,24].

## 6. Conclusions

In a referral hospital in Côte d’Ivoire, neonatal resuscitation knowledge of healthcare staff was increased after a HBB course but the performance was lower at the three-month assessment than immediately after the course. Further research should include longer follow-up periods (such as 6–12 months) to better understand the durability of training effects and to identify the optimal timing for refresher training. Moreover, future educational interventions may target other areas of improvement such as first minute priority, recognition of healthy newborn, consequences of unnecessary suctioning, bag-mask ventilation, newborn’s heart rate and post-resuscitation care.

## Figures and Tables

**Figure 1:**
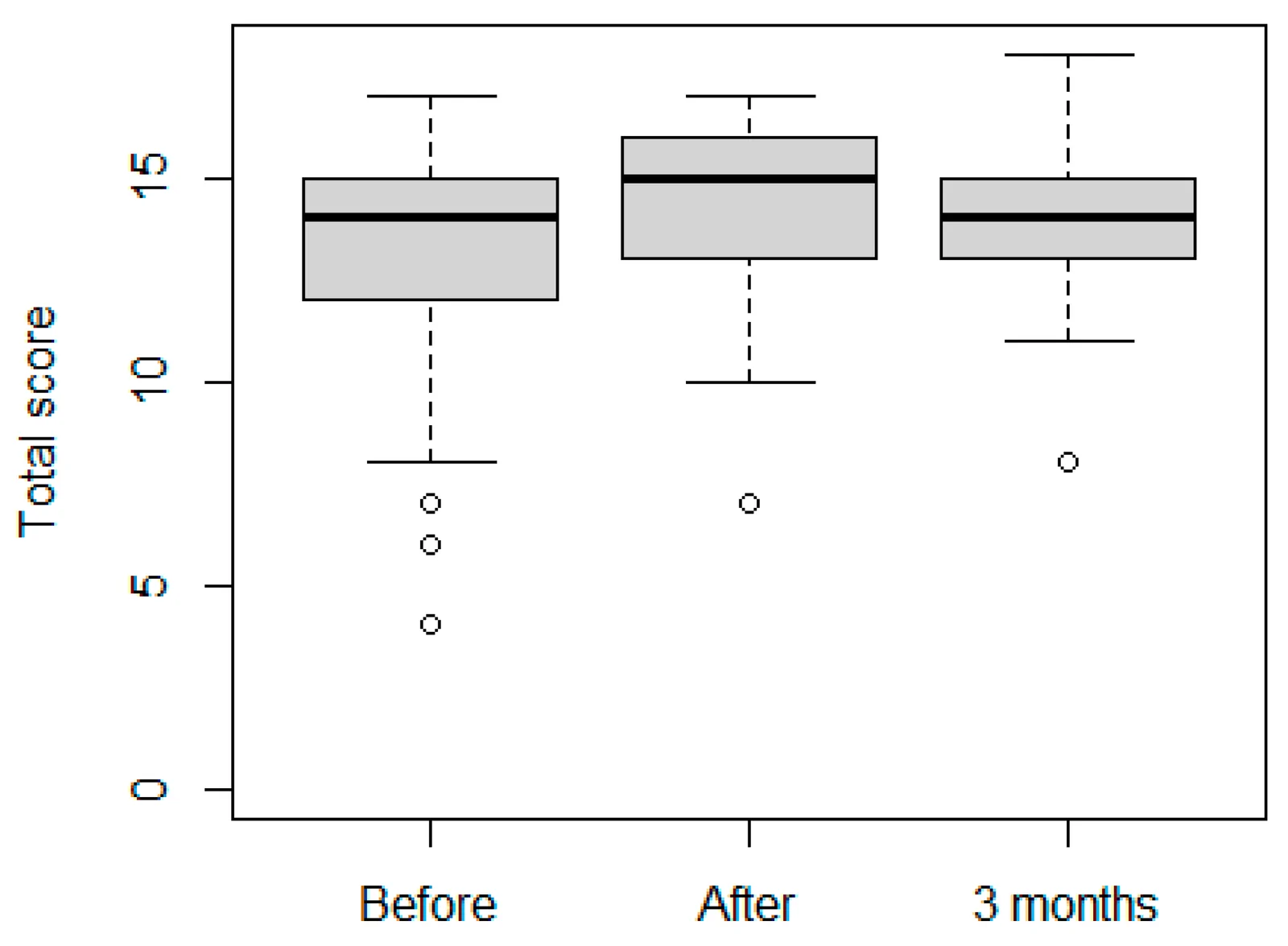
Total score at the three timepoints (before the course, after the course and three months later): box and whiskers plots.

**Figure 2:**
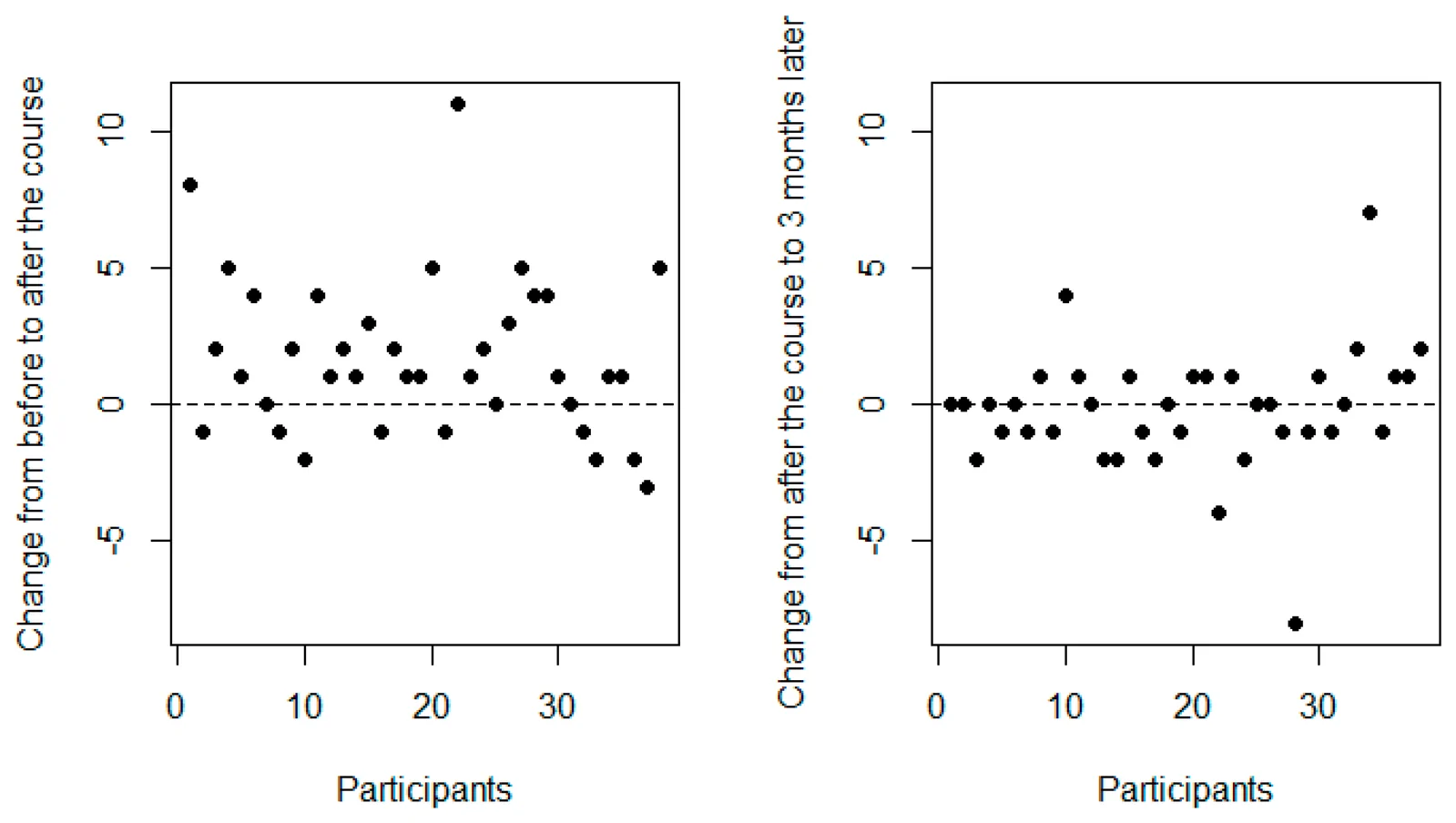
Change in the total score from before the course to after the course (**left panel**) and from after the course to three months later (**right panel**): scatter plots.

**Figure 3:**
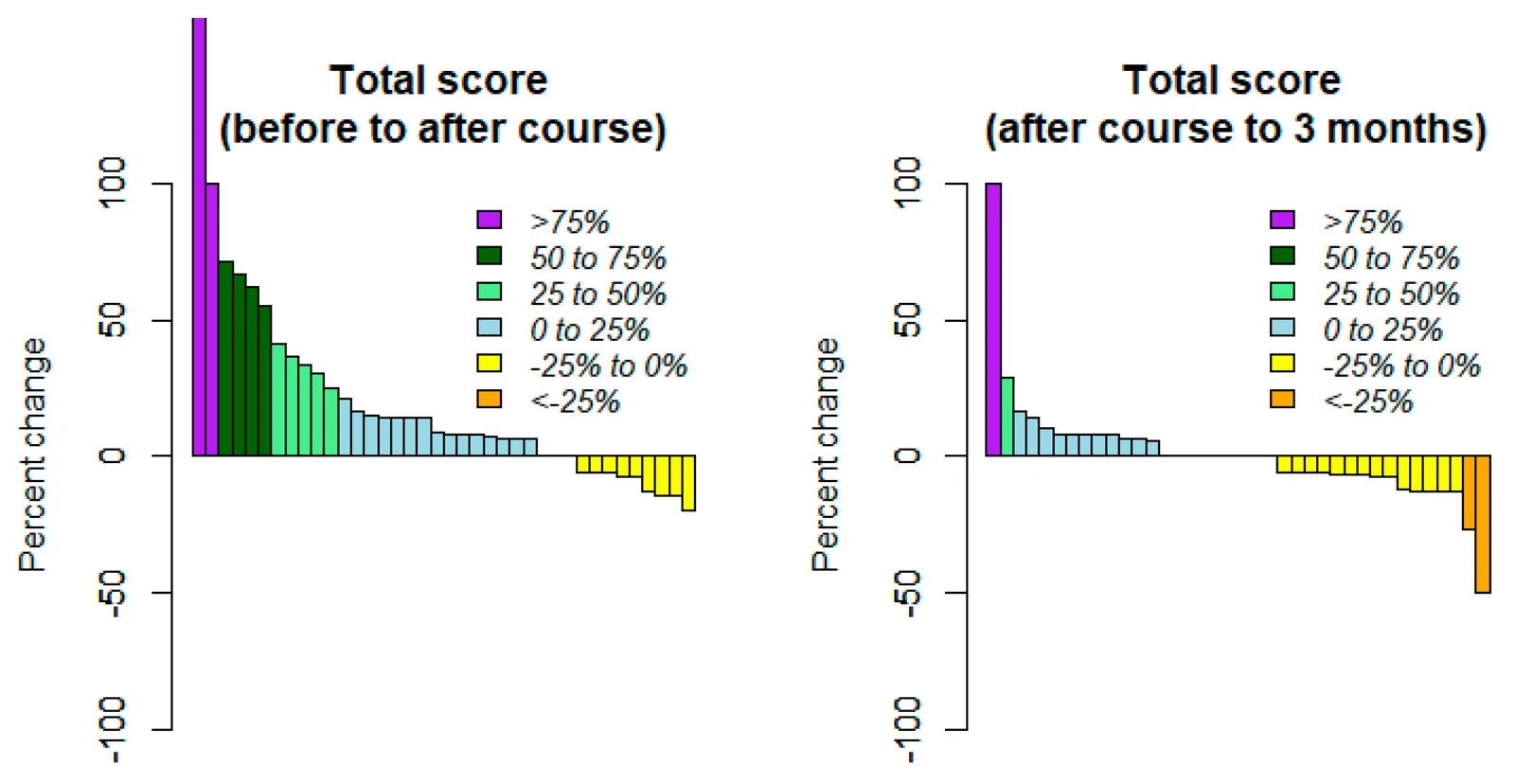
Percent change in the total score from before to after the course (**left panel**) and from after the course to three months later (**right panel**): individual waterfall plots.

**Figure 4:**
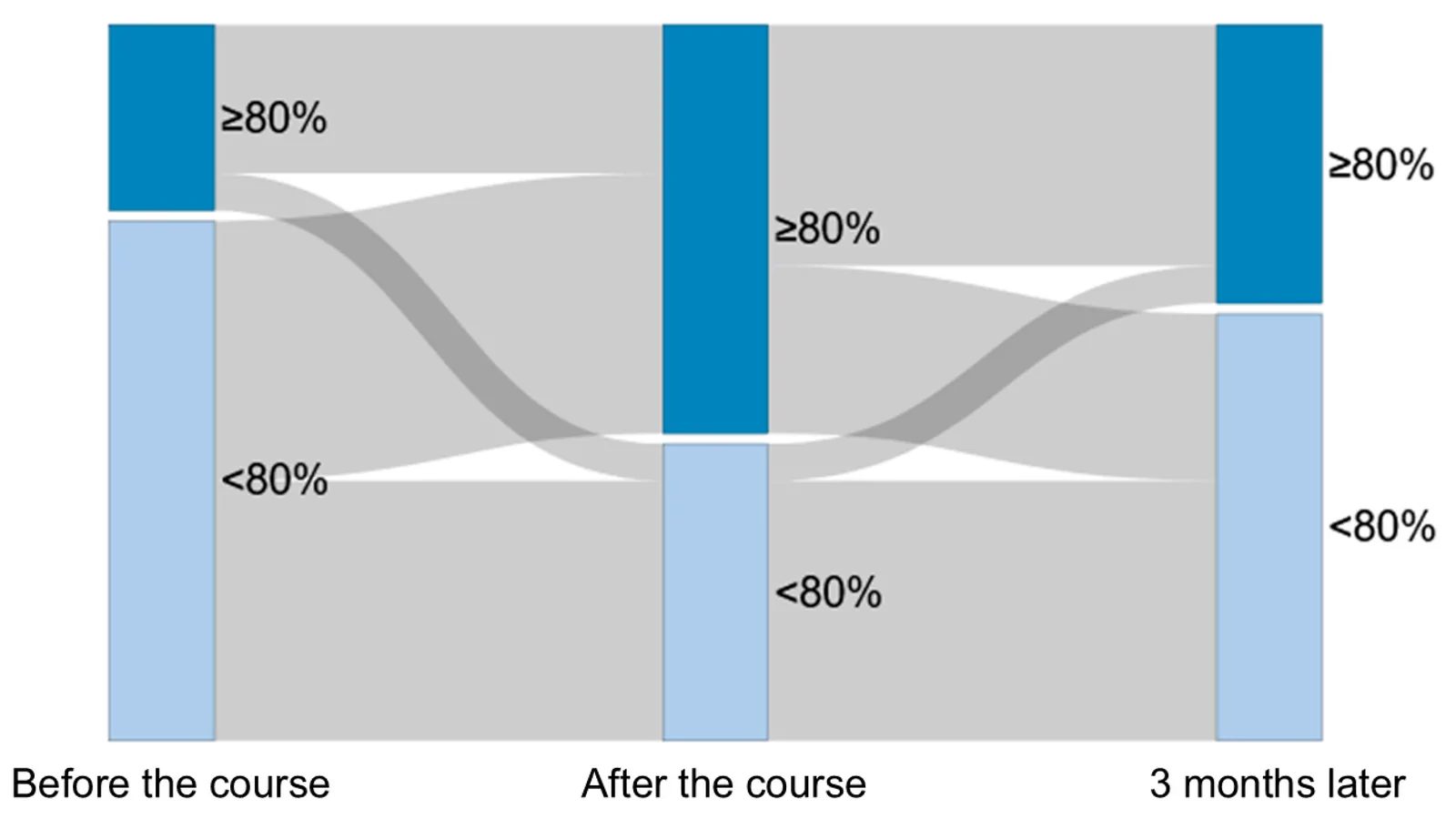
Passing criterion of ≥80% achieved by the participants before the course, after the course and three months later: Sankey diagram. The line widths are proportional to the number of participants in each flow.

**Figure 5:**
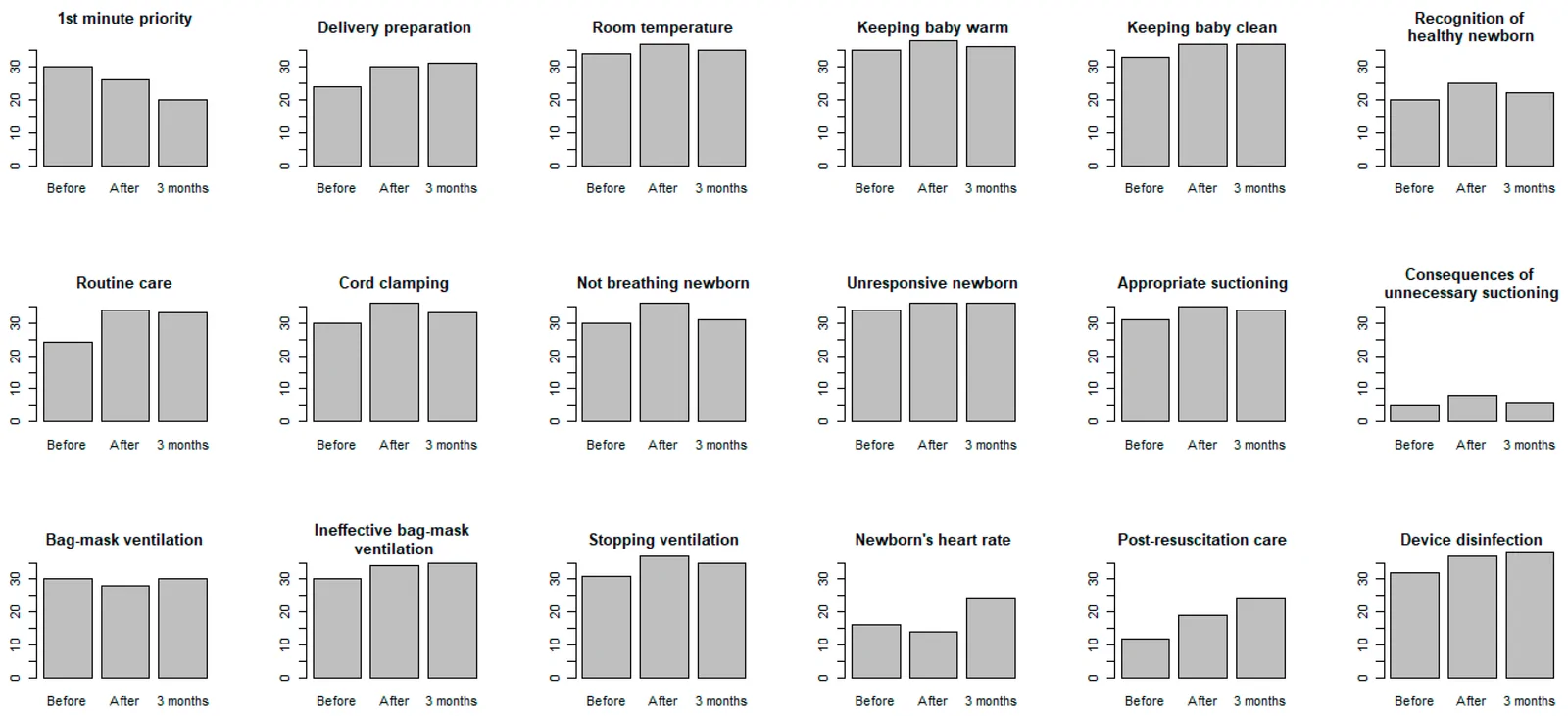
Number of correct answers for each item at the three timepoints (before the course, after the course and three months later): bar-plots. Item-level comparisons were presented only as descriptive and hypothesis-generating.

## Data Availability

The datasets used and/or analyzed are available from the corresponding author on reasonable request.

## Supplementary Materials

The following supporting information can be downloaded at: https://www.mdpi.com/article/10.3390/children13101286/s1.

## Abbreviations

CHR	Centre Hospitalier Régional Félix Houphouët-Boigny
HBB	Helping Babies Breathe
IQR	interquartile range
