# Supplementary material for: Knowledge Improvement After a Neonatal Resuscitation Course in a Low-Resource Setting
Source: Children (Basel). 2026 Sep 22;13(10):1286. doi: 10.3390/children13101286 (PMC13703640; doi:10.3390/children13101286)
Supplement: Supplementary file 1 [file children-13-01286-s001.zip › children-4515532-supplementary.pdf]

**SUPPLEMENTARY MATERIAL**

Supplementary Table S1. A multiple-choice written test was administered before and after the course to assess knowledge acquisition (September 2025), and three months later to assess knowledge retainment (December 2025). The test included 18 items and was derived from the Knowledge Check (HBB 2nd Edition), which is a validated standardized questionnaire of the HBB program.

| Item | Answers |
|---|---|
| 1. What should be done during the Golden Minute? | a. Bathe the baby<br>b. Deliver the placenta<br>c. Assess the heart rate<br>d. Help the baby breathe if necessary |
| 2. Preparing for delivery: | a. Identify an assistant and review the emergency plan<br>b. Ask everyone except the mother to leave the room<br>c. Prepare equipment only when necessary<br>d. Do not require an assistant |
| 3. To prepare the birthing area: | a. Open all doors and windows to let in fresh air<br>b. Darken the room<br>c. Ensure the area is clean, warm, and well-lit<br>d. Keep the room temperature cold |
| 4. What should be done to keep the baby warm? | a. Open all windows<br>b. Bathe the baby immediately after birth<br>c. Place hot water bottles directly against the baby's skin<br>d. Place the baby skin-to-skin with the mother |
| 5. What should be done to keep the baby clean? | a. Wash hands before touching the baby and help the mother wash her hands before breastfeeding<br>b. Reuse the suction device before cleaning it<br>c. Keep the umbilical cord well covered<br>d. Do not touch the baby |
| 6. Which baby can receive routine care after birth? | a. A baby who is not breathing<br>b. A baby who is gasping<br>c. A baby who is crying and/or breathing well<br>d. A limp baby |
| 7. Routine care for a healthy baby at birth includes: | a. Drying, removing wet linens, and bathing the baby<br>b. Drying, removing wet linens, and placing the baby skin-to-skin<br>c. Bathing and dressing the baby in clean clothes<br>d. Drying and wrapping the baby in wet linens |
| 8. When should the umbilical cord be clamped or tied and cut during routine care? | a. After delivery of the placenta<br>b. Approximately 1 to 3 minutes after birth<br>c. Immediately after birth<br>d. Before the baby has cried |
| 9. A baby is silent, limp, and not breathing at birth. What should be done? | a. Thoroughly dry the baby<br>b. Shake the baby<br>c. Splash cold water on the face<br>d. Hold the baby upside down |
| 10. A newborn is silent, limp, and not crying. The baby does not respond to respiratory | a. Slap the baby's back<br>b. Hold the baby upside down |

| | |
|---|---|
| stimulation maneuvers. What should be done next? | c. Press on the baby's ribs<br>d. Begin ventilation |
| 11. In which situation should a baby be suctioned? | a. When the baby cries at birth<br>b. When the baby cries but there is meconium in the amniotic fluid<br>c. When secretions are seen obstructing the mouth and nose<br>d. Before drying the baby |
| 12. Suctioning a baby unnecessarily or too frequently can | a. Cause the baby to stop breathing<br>b. Cause the baby to cough and gasp<br>c. Stimulate the baby to cry<br>d. Increase the baby's heart rate |
| 13. Which of the following statements about bag-and-mask ventilation is TRUE? | a. The mask must cover the eyes<br>b. Air must escape between the mask and the face<br>c. Squeeze the bag to produce slight chest movement<br>d. Squeeze the bag to deliver 80 to 100 breaths per minute |
| 14. The baby's chest is not moving during bag-and-mask ventilation. What should be done? | a. Stop ventilation<br>b. Reposition the mask to achieve a better seal<br>c. Slap the baby's back<br>d. Give the baby medication |
| 15. When can ventilation be stopped? | a. If the baby is blue and limp<br>b. If the baby's heart rate is low<br>c. If the heart rate is normal but the chest is not moving<br>d. If the heart rate is normal and the baby is breathing or crying |
| 16. A newborn's heart rate should be: | a. Faster than your heart rate<br>b. Slower than your heart rate<br>c. Checked before drying the baby<br>d. Checked only when the baby is crying |
| 17. A baby who has received ventilation: | a. Needs continuous observation alongside the mother<br>b. Cannot be fed<br>c. Always requires advanced care<br>d. Must immediately receive antibiotics |
| 18. When should the bag and mask, as well as the suction device, be disinfected? | a. After each use<br>b. Only when they appear dirty<br>c. Once a week<br>d. Once a month |

Supplementary Table S2. Number of correct answers for each question at the three timepoints (before the course, after the course and 3 months later)

| Item | Topic | Before the course | After the course | p-value | Three months after the course | p-value |
|---|---|---|---|---|---|---|
| #1 | 1st minute priority | 30 (79%) | 26 (68%) | 0.45 | 20 (53%) | 0.23 |
| #2 | Delivery preparation | 24 (63%) | 30 (79%) | 0.13 | 31 (82%) | 0.99 |
| #3 | Room temperature | 34 (89%) | 37 (97%) | 0.24 | 35 (92%) | 0.48 |
| #4 | Keeping baby warm | 35 (92%) | 38 (100%) | 0.24 | 36 (95%) | 0.48 |
| #5 | Keeping baby clean | 33 (87%) | 37 (97%) | 0.13 | 37 (97%) | 0.99 |
| #6 | Recognition of healthy newborn | 20 (53%) | 25 (66%) | 0.68 | 22 (58%) | 0.50 |
| #7 | Routine care | 24 (63%) | 34 (89%) | 0.08 | 33 (87%) | 0.99 |
| #8 | Cord clamping | 30 (79%) | 36 (95%) | 0.13 | 33 (87%) | 0.24 |
| #9 | Not breathing newborn | 30 (79%) | 36 (95%) | 0.22 | 31 (82%) | 0.37 |
| #10 | Unresponsive newborn | 34 (89%) | 36 (95%) | 0.99 | 36 (95%) | 0.99 |
| #11 | Appropriate suctioning | 31 (82%) | 35 (92%) | 0.48 | 34 (89%) | 0.99 |
| #12 | Consequences of unnecessary suctioning | 5 (13%) | 8 (21%) | 0.99 | 6 (16%) | 0.37 |
| #13 | Bag-mask ventilation | 30 (79%) | 28 (74%) | 0.18 | 30 (79%) | 0.99 |
| #14 | Ineffective bag-mask ventilation | 30 (79%) | 34 (89%) | 0.99 | 35 (92%) | 0.99 |
| #15 | Stopping ventilation | 31 (82%) | 37 (97%) | 0.61 | 35 (92%) | 0.61 |
| #16 | Newborn's heart rate | 16 (42%) | 14 (37%) | 0.18 | 24 (63%) | 0.009 |
| #17 | Post-resuscitation care | 12 (32%) | 19 (50%) | 0.45 | 24 (63%) | 0.42 |
| #18 | Device disinfection | 32 (84%) | 37 (97%) | Could not be calculate | 38 (100%) | 0.99 |
